# Immunotherapy of brain metastases: breaking a “dogma”

**DOI:** 10.1186/s13046-019-1426-2

**Published:** 2019-10-17

**Authors:** Anna Maria Di Giacomo, Monica Valente, Alfonso Cerase, Maria Fortunata Lofiego, Francesca Piazzini, Luana Calabrò, Elisabetta Gambale, Alessia Covre, Michele Maio

**Affiliations:** 1 Department of Oncology, Center for Immuno-Oncology, Medical Oncology and Immunotherapy, University Hospital of Siena, Viale Bracci, 14, 53100 Siena, Italy; 2 Unit of Neuroradiology, University Hospital, Siena, Italy

**Keywords:** Brain metastases, Immune checkpoint(s), Cancer immunotherapy, Lung cancer, Magnetic resonance imaging, Melanoma, Neuroradiology, Tumor microenvironment

## Abstract

Until very few years ago, the oncology community dogmatically excluded any clinical potential for immunotherapy in controlling brain metastases. Therefore, despite the significant therapeutic efficacy of monoclonal antibodies to immune check-point(s) across a wide range of tumor types, patients with brain disease were invariably excluded from clinical trials with these agents. Recent insights on the immune landscape of the central nervous system, as well as of the brain tumor microenvironment, are shedding light on the immune-biology of brain metastases.

Interestingly, retrospective analyses, case series, and initial prospective clinical trials have recently investigated the role of different immune check-point inhibitors in brain metastases, reporting a significant clinical activity also in this subset of patients. These findings, and their swift translation in the daily practice, are driving fundamental changes in the clinical management of patients with brain metastases, and raise important neuroradiologic challenges. Along this line, neuro-oncology undoubtedly represents an additional area of active investigation and of growing interest to support medical oncologists in the evaluation of clinical responses of brain metastases to ICI treatment, and in the management of neurologic immune-related adverse events.

Aim of this review is to summarize the most recent findings on brain metastases immunobiology, on the evolving scenario of clinical efficacy of ICI therapy in patients with brain metastases, as well as on the increasing relevance of neuroradiology in this therapeutic setting.

## Background

The occurrence of brain metastases in solid tumors is steadily increasing [1]. About 50% of cancer patients will experience metastatic spreading to the central nervous system (CNS) in the course of their disease [2–4], with the highest incidence been reported in melanoma (28.2%), lung (26.8%), renal (10.8%), and breast cancer (7.6%) [5]. The prognosis and survival of patients with brain metastases remains poor; relevant prognostic factors include age, primary disease control, presence of extracranial metastases or leptomeningeal disease, and performance status, though their clinical value is limited [6]. The overall 2 and 5 year survival estimates for patients who develop brain metastases across different tumor types are 8.1 and 2.4%, respectively, and disease spreading to the CNS represents the cause of death in more than half of these subjects [6]. Therapeutic options for patients with brain metastases are largely palliative and include surgical resection, whole-brain radiation therapy (WBRT), stereotactic radiosurgery (SRS), or their combinations [4], while chemotherapy is uncommonly utilized due to its acknowledged limitation to effectively cross the blood-brain barrier [1]. This latter notion, and the poorer prognosis of patients with brain metastases has led them to be generally excluded from clinical trials with chemotherapeutic agents in the past; a similar scenario applied also more recently to immunotherapy with immune check-point inhibitors (ICI) [7]. However, in the last years, many scientific efforts were directed to the study of the interactions between immune system and tumor microenvironment (TME) in brain metastases allowing to identify CNS as an immunologically distinct rather than an immune-isolated compartment [8]. The inflammatory TME of brain metastases has shown to be active in the majority of patients with dense infiltration of tumor-infiltrating lymphocytes (TIL) often expressing immunosuppressive factors like programmed death-1 (PD-1) ligand (PD-L1) [9]. These notions and the recent availability of effective immunotherapeutic agents [10, 11], including anti-cytotoxic T lymphocyte-associated antigen-4 (CTLA-4), anti- PD-1, and PD-L1 monoclonal antibodies (mAbs), have supported their use, also in patients with brain metastases, as well as in primary CNS tumors [12].

In this manuscript we focus on the upcoming clinical evidence demonstrating the effectiveness of immunotherapy with ICI in brain metastases, and on the daily practice implications of these finding. Lastly, we highlight potential future avenues for a more effective immunotherapeutic approach for the treatment of brain metastases.

### Brain tumor microenvironment immunobiology

The tumor microenvironment (TME) of metastatic CNS malignancies, with its highly complex cancer-promoting features, is considered among the main regulators of the response and resistance to treatment [13]. Other than endothelial cells, the brain TME consists of different cell types including fibroblasts, pericytes, microglia and astrocytes, along with a variety of immune cells with suppressive or stimulatory functions [14] physically protected by the blood-brain barrier (BBB). It was shown that the BBB in brain metastases is often compromised, not fully disrupted but rather remodeled into a blood-tumor barrier due to alterations in the pericyte subpopulation [15] encouraging a robust infiltration of multiple immune suppressive cell types from the peripheral circulation [8]. The dynamic interactions occurring between these diverse cell types and cancer cells may contribute to the metastatic progression and may impair response to therapy. Cancer cells metastatic to the brain and astrocytes can stimulate each other directly [16] or through the release of different cytokines and inflammatory mediators, contributing to brain colonization [8]. Indeed, interleukin (IL) -8, macrophage migration inhibitory factor (MIF), and plasminogen activator inhibitor-1 (PAI-1) released by metastatic lung cancer cells were found to be able to activate astrocytes that produced growth factors [i.e., IL-6, IL-1β, and tumor necrosis factor-α (TNF-α)], thus fostering cancer cell growth in the brain niche [17]. Moreover, in vitro studies demonstrated that neurotrophic factors secreted by reactive astrocytes such as IL-6, transforming growth factor-β (TGF-β), insulin-like growth factor-1 (IGF-1), and chemokine ligand 12a (CXCL12a) may contribute to the development of brain metastasis from breast cancer [18, 19]. In addition, brain-metastasizing melanoma cells were found to reprogram astrocytes to express the pro-inflammatory cytokine IL-23, which stimulated the secretion of matrix metalloproteinase-2 (MMP-2) enhancing the degradation of the extracellular matrix, and facilitating the extravasation and eventually brain invasion by tumor cells [20].

Besides “resident” astrocytes, type 2 tumor-associated macrophages [21], myeloid-derived suppressor cells (MDSC), regulatory T cells (T-reg) [14], and cancer-associated fibroblasts (CAF) with pro-tumorigenic characteristics were found to be recruited in the brain by metastatic melanoma, breast and colon cancer [22, 23]. These different cell types were shown to play a negative role in the anti-tumor immune response by reducing the expression of key molecules involved in T-cell co-stimulation (e.g., CD80, CD86, CD40) [8], impairing antigen presentation [24], and deregulating the homeostasis of the brain microenvironment [8]. In this highly suppressive TME metastatic landscape, TIL are poorly represented and functionally impaired in brain metastases, compared to primary tumors [25]. Along this line, different studies documented a down-regulated T-cell activity as the result of tumor-induced T cell exhaustion in brain metastasis; indeed, PD-1 expression was detected on > 60% of TIL [16], though a correlation with clinical outcome remains to be investigated.

Besides the analyses of the different cell population in the brain metastases TME, controversial results have been reported on the exclusive brain metastases molecular profiles. Although several studies demonstrated a genetically divergence [e.g., higher rates of BRAF mutations, higher tumor mutational burden (TMB), higher PD-L1 expression, private gene mutations] between brain metastases and their primary tumors [26–28], no significant differences were observed in mutation profiles between a case series of breast cancer brain metastases and their primary lesions [29]. These findings suggested that additional studies are required to fully identify the unique molecular characteristics/features of brain metastases.

The immunosuppressive role of TME of CNS metastases highlights the need for new therapeutic approaches promoting M1 properties of macrophages, the recruitment of tumor infiltrating CD8 + T cell [30], or targeting suppressive cell types such as T-reg and MDSC. Along this line, it has been demonstrated that the coadministration of a Treg-depleting anti-CD25 mAb and IL-21-engineered cell vaccine led to the cure of most mice bearing TS/A micrometastases [31].. Moreover, strategies aiming at converting the immune-suppressive milieus into inflamed environments [31, 32] through the use of antibodies against suppressive cytokines such as TGF-β, or IL-10 may play a relevant role [33]. In this context, the overexpression of TGF-β2 by melanoma cells was associated with site-specific brain metastasis; and, accordingly, the use of anti-TGF-β2 therapies in a syngeneic murine melanoma model significantly reduced metastasis to the brain [34].

Upcoming findings suggest that epigenetic modeling may also contribute to the immune-suppressive tumor and TME profile of brain metastases. Initial evidence supporting this hypothesis derived from the exploration of the methylomes of lung, breast, and cutaneous melanoma brain metastases, and from their relative primary tumors. These studies allowed building epigenetic classifiers able to determine the origin of brain metastases, the histotype of primary CNS tumors, and also the therapeutic subtype for breast cancer patients [34]. Consistent with the notion that epigenetic modeling plays a relevant role in shaping brain metastases, genes involved in cell development and differentiation, regulation of gene expression, cell migration, and tumor suppression were found to be unmethylated in the majority of breast cancer patients without brain metastases as compared to those with CNS involvement [35]. These findings, and the well-known immunomodulatory potential of DNA hypomethylating agents on genes involved antigen processing and presentation [36], and on immunosuppressive cellular and soluble components of the TME of brain metastases (e.g., MDSC, T-reg, chemokines) [37, 38], strongly support the use of epigenetic drugs combined with ICI to develop new strategies for the personalized therapeutic management of patients with brain metastases.

### ICI therapy of brain metastases

#### Melanoma

The initial clinical evidence of ICI activity in melanoma brain metastases was generated in two prospective phase II studies. The first trial investigated the efficacy of ipilimumab in patients with asymptomatic (*n* = 51, cohort A) or symptomatic (*n* = 21, cohort B) brain metastases [39]. CNS disease control rate (DCR) at 12 weeks was 24 and 10%, and the intracranial overall response rate (ORR) was 16 and 5%, in cohorts A and B, respectively; median overall survival (OS) was 7 months (range 0.4–31+) for cohort A, and 4 months (0.5–25+) for cohort B, while survival rates at 24 months were 26, and 10%, respectively. Though initial, these results suggested a better efficacy of treatment in patients with asymptomatic brain metastases and who did not receive steroids. In the phase II Italian Network for Tumor Biotherapy (NIBIT)-M1 study, 86 patients with metastatic melanoma received ipilimumab at 10 mg/kg combined with fotemustine: among the 20 patients who also had asymptomatic brain metastases at study enrolment the ir-DCR was 50%, and it was 46.5% in the whole population [40]. Also, the 3 year OS was 27.8% in patients with brain metastases and 28.5% in the whole population, suggesting a durable clinical benefit of treatment also in patients with asymptomatic brain metastases [41]. A more recent follow-up of this study has shown that 5 complete regressions of brain disease were obtained, with a duration of brain complete response (CR) of 16, 28, 39, 80+, 94+ months; of note, the 2 patients still alive, in the absence of subsequent treatment, had achieved a CR both intra- and extra-cranial (A.M. Di Giacomo, et al. unpublished). Based on this intriguing clinical evidence and on available results showing an additive therapeutic efficacy of ipilimumab combined with nivolumab in melanoma [42], the multicentre, phase III, randomized, open-label NIBIT-M2 study, sponsored by the NIBIT Foundation, was activated [43]. This three-arm study was designed to assess the OS of previously untreated metastatic melanoma patients with asymptomatic brain metastases who received fotemustine, its combination with ipilimumab, or the combination of ipilimumab and nivolumab. Results from a pre-planned interim analysis of the study will be soon available.

Providing additional support to the notion that patients with brain metastases can benefit from ICI treatment, the activity of anti-PD-1 monotherapy was recently reported in a retrospective analysis of 66 melanoma patients with CNS disease treated with nivolumab or pembrolizumab [44]. An intracranial ORR and DCR of 21 and 56%, respectively, with a median OS of 9.9 months was observed [44]. Moreover, in a prospective phase II study, pembrolizumab induced in 23 melanoma patients an intracranial ORR of 26%, with 2 partial responses (PR) and 4 CR. With a median follow-up of 24 months the median progression free survival (PFS) and OS were 2 and 17 months, respectively, and 11 patients (48%) were still alive at 2 years [45].

Two additional studies have recently investigated the dual blockade of CTLA-4 and PD-1 molecules in melanoma patients metastatic to the brain. The phase II, single-arm, CheckMate 204 study enrolled patients with asymptomatic brain metastases measuring 0.5–3.0 cm, that were treated with a combination of ipilimumab and nivolumab for 4 cycles, followed by nivolumab maintenance until progression or unacceptable toxicity [46]. Among the 94 enrolled patients the intracranial and extracranial ORR were 55 and 50%, respectively, with a global ORR of 51%, and with 90% ongoing objective responses at a relatively short median duration of follow-up of 14 months [46]. A recent update of the study with a median follow-up of 20.6 months, reported an intracranial and extracranial ORR of 54 and 49%, respectively, with a global ORR of 51%, among the 101 evaluable patients with asymptomatic brain metastases; the 18 months survival rate was 75% [47]. Noteworthy, results from a cohort of 18 patients with symptomatic brain metastases demonstrated an intracranial, extracranial, and global ORR of 22%, with a 6 months survival rate of 66% at a median follow-up of 5.2 months [47]. Consistent with these results are those from the Australian Brain Collaboration (ABC) study, a phase II, prospective trial enrolling 3 cohorts of patients with asymptomatic or symptomatic brain metastases [48]. Subjects with no prior local brain treatment were randomly assigned to receive nivolumab combined with ipilimumab (Cohort A) or nivolumab alone (Cohort B), whereas symptomatic patients who had failed local brain therapy and/or had leptomeningeal spreading disease received nivolumab alone (Cohort C). The intracranial ORR was 46, 20, and 6% in Cohorts A, B and C, respectively. Among patients enrolled in Cohort A, those with treatment-naïve brain disease achieved a 56% ORR while it was 16% in BRAF mutant patients pre-treated with BRAF and MEK inhibitors [48]. Corroborating the safety results from CheckMate 204 study, treatment-related grade 3/4 adverse events occurred in 19 patients (54%) in Cohort A, in one patient (4%) in Cohort B, and in two patients (13%) in Cohort C, with no unexpected toxicities; these findings supported the safety and tolerability of nivolumab alone or in combination with ipilimumab in melanoma patients with brain metastases [48].

Overall, data from these prospective clinical trials demonstrate safety and efficacy of anti-CTLA-4 plus anti-PD-1 therapy, coupled with important ORR, similar to those reported in extracranial sites. The findings are highly encouraging and strongly support the role of ICI therapy also in patients with brain metastases (Table 1). Importantly, additional therapeutic combinations in melanoma patients with brain metastases are being explored, as summarized in Table 2.

**Table 1:** Efficacy of immune checkpoint inhibitors in melanoma brain metastases

Author	Phase	Agent	No. Patients	Intracranial ORR (%)
*Margolin (2012)*	II	Ipi		
Cohort A			51	16
Cohort B			21	5
*Di Giacomo (2012)*	II	Ipi + Fotemustine	20	50
*Parakh (2017)*	Real-world (retrospective)	Nivo or Pembro	66	21
*Kluger (2019)*	II	Pembro	23	26
*Long (2017)*	II			
Cohort A		Ipi + Nivo	26	46
Cohort B		Nivo	25	20
Cohort C		Nivo	16	6
*Tawbi (2018)*	II	Ipi + Nivo	94	55

*Ipi* Ipilimumab, *Nivo* Nivolumab, *Pembro* Pembrolizumab, *ORR* Object Response Rate

**Table 2:** Summary of ongoing clinical trials with ICI in solid tumor with brain metastases^a^

Clinical trial identifier	Trial Name	Phase	Status
NCT03175432	Study of Bevacizumab in Combination With Atezolizumab in Patients With Untreated Melanoma Brain Metastases (BEAT-MBM)	II	Recruiting
NCT02460068	A Study of Fotemustine (FTM) Vs FTM and Ipilimumab (IPI) or IPI and Nivolumab in Melanoma Brain Metastasis (NIBIT-M2)	III	Recruiting
NCT03340129	Anti-PD 1 Brain Collaboration + Radiotherapy (ABC-X Study) (ABC-X)	II	Not yet recruiting
NCT03728465	Evaluation of Safety and Efficacy of Patients With Four and More Symptomatic Brain Metastases of Melanoma	II	Recruiting
NCT02681549	Pembrolizumab Plus Bevacizumab for Treatment of Brain Metastases in Metastatic Melanoma or Non-small Cell Lung Cancer	II	Recruiting
NCT02858869	Pembrolizumab and Stereotactic Radiosurgery for Melanoma or Non-Small Cell Lung Cancer Brain Metastases	I	Recruiting
NCT03563729	Melanoma Metastasized to the Brain and Steroids (MEMBRAINS)	II	Recruiting
NCT03873818	Low Dose Ipilimumab With Pembrolizumab in Treating Patients With Melanoma That Has Spread to the Brain	II	Recruiting
NCT02130466	A Study of the Safety and Efficacy of Pembrolizumab (MK-3475) in Combination With Trametinib and Dabrafenib in Participants With Advanced Melanoma (MK-3475-022/KEYNOTE-022) *(patients with inactive brain metastases eligible)*	I/II	Recruiting
NCT02696993	Nivolumab and Radiation Therapy With or Without Ipilimumab in Treating Patients With Brain Metastases From Non-small Cell Lung Cancer	I/II	Recruiting
NCT02978404	Combining Radiosurgery and Nivolumab in the Treatment of Brain Metastases	II	Recruiting
NCT02886585	Pembrolizumab In Central Nervous System Metastases	II	Recruiting
NCT03867175	Immunotherapy With or Without SBRT in Patients With Stage IV Non-small Cell Lung Cancer	III	Not yet Recruiting
NCT03719768	Avelumab With Radiotherapy in Patients With Leptomeningeal Disease	I	Recruiting
NCT03325166	Pembrolizumab and Magnetic Resonance Imaging With Ferumoxytol in Treating Patients With Non-small Cell Lung Cancer and Brain Metastases	II	Recruiting
NCT02648477	Pembrolizumab and doxorubicin hydrochloride or anti-estrogen therapy in treating patients with triple-negative or hormone receptor-positive metastatic breast cancer	II	Recruiting
NCT03483012	Atezolizumab + Stereotactic Radiation in Triple-negative Breast Cancer and Brain Metastasis	II	Recruiting
NCT03449238	Pembrolizumab And Stereotactic Radiosurgery (SRS) Of Selected Brain Metastases In Breast Cancer Patients	I/II	Recruiting
NCT03526900	Atezolizumab in Combination With Carboplatin Plus Pemetrexed in Chemotherapy-naïve Patients With Asymptomatic Brain Metastasis (ATEZO-BRAIN)	II	Recruiting

^a^ as of Sep 4, 2019. Source: clinicaltrials.gov

*ABC* Australian Brain Collaboration, *SRS/SBRT* stereotactic radiosurgery

#### Lung Cancer

As it had previously occurred for melanoma, patients with non-small cell lung cancer (NSCLC) and active brain metastases were excluded from pivotal clinical trials with ICI, and only a few retrospective analyses have presently investigated the efficacy and safety of ICI therapy in this patient population. In a prospective phase II trial pembrolizumab induced an intracranial ORR in 10 out of 34 (29.4%) PD-L1^+^ patients, with no objective response in the 5 PD-L1^−^ patients treated. The median OS among all patients was 8.9 months, and 31% of patients were alive at 2 years [49]. A pooled analysis from the three CheckMate studies 063 (phase II), 017 (phase III), and 057 (phase III), explored the role of nivolumab in NSCLC patients with previously treated or untreated asymptomatic brain metastases [50]. Among evaluable patients with pre-treated brain metastases at the time of overall disease progression (PD) or last tumor assessment, 33% had no evidence of CNS progression while 52% had progressive brain disease; median OS was longer in the nivolumab group (8.4 months) as compared to the chemotherapy (docetaxel) group (6.2 months). Supporting the efficacy of ICI in NSCLC patients with brain metastases, the Italian expanded access program (EAP) with nivolumab enrolled 409 patients with asymptomatic or pretreated brain metastases who achieved an ORR of 17% and a DCR of 40% [51].

In addition, an exploratory subgroup analysis of the OAK study [52], assessing the safety and efficacy of the anti-PD-L1 atezolizumab in patients with or without a history of asymptomatic, treated brain metastases, has shown an acceptable safety profile with a trend toward an OS benefit of atezolizumab versus docetaxel (16 versus 11.9 months). Interestingly, atezolizumab led to a prolonged time to radiologic identification of new symptomatic brain metastases compared with docetaxel [53].

Aiming to expand these initial intriguing observations, supporting the role of immunotherapy also in lung cancer patients with brain disease, several ongoing prospective clinical trials are investigating the efficacy and safety of ICI in NSCLC and small cell lung cancer (SCLC) patients with brain metastases (Table 2). Moreover, initial studies aim to explore the role of new prognostic and predictive biomarkers also in NSCLC with brain metastases [54, 55].

#### Renal cell carcinoma

The 5 year cumulative incidence of brain metastases in renal cell carcinoma (RCC) ranges from 7 to 13% [56], and limited data are available on the efficacy of current systemic treatment of brain disease in RCC patients. To date the vast majority of prospective trials in RCC allowed the inclusion of patients with stable brain disease, and none of the pivotal trials with ICI reported the efficacy of the immunotherapy in patients with active brain metastases. Initial signs of clinical activity for ICI therapy in brain metastases from RCC derived from case reports and small observational series. Among the latter, the Italian EAP with nivolumab enrolled 389 patients beyond first-line therapy, of whom 32 (8%) had asymptomatic brain metastases that did not require radiotherapy or high dose steroids (i.e., > 10 mg of prednisone). The 6 and 12 months survival rates of these patients were 87 and 66.8%, and they were 80.0 and 63.1% in the overall population; the DCR was 53.1 and 53.0% in patients with or without brain metastases, respectively. Treatment related adverse events (AE) were similar between patients with CNS metastases and the overall population (31% vs 32%); however, grade 3–4 toxicities were more frequent (12% vs 7%) in patients with brain disease [57]. The French phase II study GETUG-AFU 26 NIVOREN also evaluated safety and efficacy of nivolumab in metastatic RCC after progression on vascular endothelial growth factor receptor (VEGFR)-directed therapies [58]. Seventy-three patients with asymptomatic brain metastases were treated: Cohort A included 39 patients who had not received any prior brain therapy (i.e., surgery, radiation, steroids) while Cohort B included 34 patients who had received prior local treatment (primarily SRS). The primary endpoint was the intracranial ORR in Cohort A according to modified Response Evaluation Criteria in Solid Tumors (RECIST) criteria, allowing target lesions ≥5 mm. Intracranial ORR was 12% in Cohort A but no objective responses were observed in patients with multiple brain lesions or larger than 1 cm. At a median follow up of 23.6 months median duration of treatment was 4.9 months in Cohort A, with 13% of patients still on therapy at the time of analysis. Interestingly, the intracranial median PFS was 2.7 months and the 12 months OS rate was 67% [58]. Additional data derived from the phase 3/4 study CheckMate 920 combining treatment with ipilimumab and nivolumab. The study enrolled 28 RCC patients with non-active brain metastases of whom 21 had non-target lesions at baseline (lesions < 10 mm or previously irradiated): the ORR was 29% with no CR and 8 PR; median PFS was 9.0 months and the 6 months survival rate was 89%. The incidence of immune-related (ir) AEs was generally consistent with the global safety profile of the combination [59]. These data, despite the limited case series, suggest for the efficacy and safety of ICI also in brain metastases from RCC, though further investigation in prospective clinical trials is needed to draw more solid conclusions on its efficacy in this subset of patients. In fact, recent evidence supports the notion that responsiveness to immunotherapy in RCC with brain disease seems to be multifactorial and heterogeneous, therefore several factors (e.g., TME components, genetic intratumoral heterogeneity, compartment- or location-specific alterations of signaling pathways) need to be further explored to improve efficacy of ICI treatment in RCC with brain metastases [60]..

Table 2 reports the ongoing clinical trial with ICI in patients with RCC metastatic to the brain.

### Neuroradiology and immunotherapy response evaluation

Optimal therapeutic management of cancer patients benefits from reliable diagnostic, prognostic and predictive imaging markers, aiming to identify successful treatment prior to changes in tumor size. In the therapeutic management of brain metastases, neuroradiology [61, 62] provides i) number, location, and size of brain metastases at diagnosis, ii) the differential diagnosis versus a wide range of pathologic conditions including primary tumors, vascular malformations, ischemia, hemorrhage, and seizure [63], iii) evaluation of treatment response, and iv) diagnosis of treatment-related AEs or complications. Magnetic resonance imaging (MRI) replaced computed tomography (CT) as the imaging modality of choice for brain metastases in the 1980s. Magnetic field, gradients, advanced sequences, hardware, and software are greatly expanding, as well as image post-processing, allowing quantitative data extraction and analysis capabilities. Positron emission tomography (PET) scanning also has advanced with the more widespread adoption of amino acid tracers replacing traditional [18F]-fluorodeoxyglucose, with improvements in signal-to-noise ratio and diagnostic sensitivity and specificity.

#### Diagnosis and differential diagnosis

Head CT is generally reserved to staging and restaging of asymptomatic patients, as well as, in the emergency setting, to rule out hemorrhage, ischemia, and hydrocephalus. Instead, MRI is undoubtedly the gold standard technique that should be utilized in all patients with malignant disease and with a clinical history suggestive for brain metastases [64]. According to the European Society for Medical Oncology guidelines, head MRI screening for brain metastases in patients with unresectable stage III or IV lung cancer is recommended, even though they are neurologically asymptomatic; additionally, head MRI should be utilized prior to curative surgery, regardless of the preoperative stage [65, 66]. Conversely, brain imaging should not be carried out routinely in asymptomatic metastatic breast cancer patients [67], and for melanoma patients there is currently no consensus on its frequency during the clinical follow-up [68]. The MRI protocol study needs unenhanced T1-weighted, fluid attenuated inversion recovery, T2-weighted, T2*-weighted or susceptibility-weighted images which clearly differentiates abnormal from normal signals of the nervous tissue. Gadolinium-enhanced T1-weighted magnetic resonance (MR) images are the mainstay of the neuroradiological evaluation of brain metastases since they are easy to perform, and accurately depict the margins of most intra-axial metastases, as well as leptomeningeal, dural-based, and bone metastases. Furthermore, non-morphological or so-called “functional” or “physiological” or “advanced” MR techniques may be useful to further differentiate brain metastases from other neoplastic and non-neoplastic lesions. Most common advanced MR techniques are diffusion weighted imaging with apparent diffusion coefficient measure, perfusion- and permeability-weighted imaging, and MR spectroscopy; however, none of these techniques alone has been proven to be highly specific [62, 69–71]. Thus, a thoughtful synthesis using a combination of these techniques can usually allow the neuroradiologist to correctly discriminate tissues.

#### Response evaluation

Available therapeutic options including surgery, radiation, chemotherapy, and most recently immunotherapy, may significantly affect the imaging features of both brain metastases and brain parenchima, resulting in a quite complex neuroradiological interpretation of post-treatment findings. Notably, the broader application of cancer immunotherapy to patients with brain disease, makes the complexity of neuroradiologic tumor response evaluation increasingly challenging for the neuroradiologist. Clinical responses occurring after initial disease progression or even after the appearance of new lesions, treatment-induced inflammation, long-term benefit and tumor regression are in fact frequent features in the course of immunotherapy.

Thus, aiming to standardize the radiological evaluation of brain metastases, the Response Assessment in Neuro-Oncology (RANO) Brain Metastases (RANO-BM) group proposed novel evaluation criteria focused on the objective measurement of tumor size at gadolinium-enhanced T1-weighted MR images, corticosteroid use, and clinical deterioration [72]. The RANO-BM were subsequently incorporated into the immunotherapy RANO (iRANO) criteria [73], providing recommendations for the interpretation of neuroradiological changes in the course of immunotherapy. Specifically, in the absence of worsening neurologic signs, the iRANO recommend a 3 months confirmation of initial PD, within 6 months from the beginning of treatment. If follow-up neuroradiology confirms disease progression, the date of actual progression should be backdated to the date of first neuroradiogical assessment of PD. Also, the appearance of new lesions 6 months or less from the beginning of immunotherapy does not define PD [73].

Of note, the iRANO criteria are limited to intra-axial brain metastases, as leptomeningeal and skull bone metastases are often more difficult to be objectively measured and followed, and thus still rely on a qualitative evaluation. Therefore, the RANO group had initially proposed a *Leptomeningeal* Assessment in Neuro-Oncology (LANO) scorecard [74] that has recently evolved in a simplified one [75].

The RANO- and iRANO-BM response assessment criteria undoubtedly provide a useful framework for a more effective communication between the neuroradiologist, the neuro-oncologist and clinicians utilizing immunotherapy. Nonetheless, it is imperative for neuroradiologists to be more comprehensively familiar with treatment response criteria and treatment-induced changes of brain lesions [62, 69–71]. Among these are radiation-induced brain injuries that comprise a wide range of neuroradiological findings resulting from fractionated or WBRT [76, 77], and include the development of pseudo-progression of disease that typically occurs within the first 3 months following therapy.

Furthermore, recent evidence suggest that ICI therapy can increase: *i*) the rates of hemorrhage of melanoma brain metastases treated with WBRT [78]; *ii*) the incidence of radiation necrosis after treatment of brain metastases with SRS [79]; *iii*) tumor pseudo-progressions which typically occur within the first 3 months following therapy and that is thought to represent a milder form of radiation necrosis [61, 80, 81]. Immunotherapy alone can also generate neuroradiological changes that may be misplaced with tumor recurrence or progression [82].

Additionally, despite their prominent role in the RANO and iRANO criteria gadolinium-enhanced T1-weighted images do not breakdown all possible changes occurring after treatment of brain metastases. Thus, non-morphological MR techniques may be useful to further differentiate residual/recurrent tumor from post-treatment changes. A thoughtful synthesis using a combination of these techniques can usually allow the neuroradiologist to correctly discriminate tumor tissues from treatment-induced alterations. Therefore, neuroradiologists need to have a thorough knowledge of available conventional and advanced techniques [62, 69–71, 83] to evaluate treatment response and potential treatment-related complications. Also, PET-based imaging, especially with aminoacid tracers, provides information on tumor metabolism and is currently under investigation to properly differentiate neoplastic tissues from non-specific, treatment-related changes occurring after surgery, radiotherapy, chemotherapy, and immunotherapy [84–87]. Recommendations on the clinical use of PET in neuro-oncology have been recently reported [84].

#### Adverse events

In addition to these multiple challenges in response evaluation of brain metastases, ICI therapy is well acknowledged to lead to ir AEs in a proportion of patients. Among these ir-hypophysitis (HP) can occur during treatment with a higher frequency in patients undergoing CTLA-4 blockade [88]. Notably, the incidence of ir-HP ranges from 0.5 to 18%, depending on the dose of anti-CTLA-4 therapy utilized, and from its combination with PD-1 blocking agents [89]; conversely, ir-HP is infrequent in patients treated with single agent PD-1/PD-L1 blockade. Despite HP must be differentiated from metastatic disease to the hypophysis (accounting for 0.87% of patients with intracranial metastases), its diagnosis is mainly “presumptive” as no surgery is usually performed. Thus, the diagnosis of HP is mostly based on the association of clinical signs and hormonal deficits and abnormalities, hyponatremia, and/or pituitary imaging abnormalities suggestive for HP. Furthermore, the pituitary gland may appear normal at first MRI, though it does not necessarily rule out HP [90].

More rare and potentially fatal ICI-mediated neurologic complications, including limbic encephalitis, aseptic meningitis, Guillain-Barré syndrome, transverse myelitis, myasthenia inflammatory myopathy, and orbital myositis, have been reported [91–93]. These uncommon toxicities and their swift diagnosis and optimal clinical management, undoubtedly necessitate a multidisciplinary team approach that must include the neuroradiologist.

## Conclusion

The forthcoming results of ICI-based therapeutic combination(s) in patients with brain disease may soon lead to significant changes in their comprehensive management, thus revisiting the role of surgery and radiotherapy in CNS metastases. Nevertheless, the efficacy of ICI therapy on brain metastases from tumors where ICI therapy is already the standard of care requires a thoughtful, case-by-case, evaluation on the optimal therapeutic approach to be pursued. In selected cases, ICI therapy alone could indeed represent the optimal therapeutic choice. In this daily practice scenario, as well as when patients with CNS metastases are enrolled in clinical trials, a multidisciplinary interaction is mandatory for their optimal management and must undoubtedly include the neuroradiologist to support treating physicians in evaluating clinical response and neurological side effects.

Upcoming insights from pre-clinical and clinical studies will also allow designing new therapeutic strategies to overcome the limitations deriving from the highly immunosuppressive TME of brain metastases.

## Data Availability

Not applicable.

## Abbreviations

ABC: Australian brain collaboration
AE: Adverse event
BBB: Blood-brain barrier
CAF: Cancer-associated fibroblast
CNS: Central nervous system
CR: Complete response
CT: Computed tomography
CTLA-4: Cytotoxic T lymphocyte-associated antigen-4
CXCL12a: Chemokine (C-X-C motif) ligand 12a
DCR: Disease control rate
EAP: Expanded access program
HP: Hypophysitis
ICI (s): Immune checkpoint(s)
IGF-1: Insulin-like growth factor-1
IL: Interleukin
IL-10: Interleukin-10
IL-1β: Interleukin-1β
IL-23: Interleukin-23
IL-6: Interleukin-6
IL-8: Interleukin-8
Ir: Immune-related
iRANO: Immunotherapy RANO
LANO: Leptomeningeal assessment in neuro-oncology
mAb: Monoclonal antibody
MDSC: Myeloid-derived suppressor cell
MIF: Macrophage inhibitory factor
MMP-2: Matrix metallopeptidase 2
MR: Magnetic resonance
MRI: Magnetic resonance imaging
NIBIT: Italian network for tumor biotherapy
NSCLC: No small cell lung cancer
ORR: Overall response rate
OS: Overall survival
PAI-1: Plasminogen activator inhibitor-1
PD: Disease progression
PD-1: Programmed death-1
PD-L1: Programmed death-ligand 1
PET: Positron emission tomography
PFS: Progression free survival
PR: Partial response
RANO: Response assessment in neuro-oncology
RANO-BM: Response assessment in neuro-oncology brain metastases
RCC: Renal cell carcinoma
RECIST: Response Evaluation Criteria in Solid Tumors
SCLC: Small cell lung cancer
SRS: Stereotactic radiosurgery
TGF-β: Transforming growth factor-β
TIL: Tumor-infiltrating lymphocyte
TMB: Tumor mutational burden
TME: Tumor microenvironment
TNF-α: Tumor necrosis factor-α
T-reg: Regulatory t cell
VEGFR: Vascular endothelial growth factor receptor
WBRT: Whole-brain radiation therapy
